# Dopamine Transporter Deficient Rodents: Perspectives and Limitations for Neuroscience

**DOI:** 10.3390/biom13050806

**Published:** 2023-05-09

**Authors:** Artem Savchenko, Giorgia Targa, Zoia Fesenko, Damiana Leo, Raul R. Gainetdinov, Ilya Sukhanov

**Affiliations:** 1 Valdman Institute of Pharmacology, Pavlov First St. Petersburg State Medical University, Lev Tolstoy Str. 6-8, 197022 St. Petersburg, Russia; savcenkoartem334@gmail.com; 2 Department of Pharmacological and Biomolecular Sciences “Rodolfo Paoletti”, Università degli Studi di Milano, Via Balzaretti 9, 20133 Milano, Italy; 3 Institute of Translational Biomedicine, St. Petersburg State University, 7/9 Universitetskaya Emb., 199034 St. Petersburg, Russia; 4 Department of Neurosciences, University of Mons, 7000 Mons, Belgium; 5 St. Petersburg University Hospital, St. Petersburg State University, Fontanka River Emb. 154, 190121 St. Petersburg, Russia

**Keywords:** dopamine transporter, hyperdopaminergia, hypodopaminergia, dopamine transporter knockout rodents, locomotor hyperactivity

## Abstract

The key element of dopamine (DA) neurotransmission is undoubtedly DA transporter (DAT), a transmembrane protein responsible for the synaptic reuptake of the mediator. Changes in DAT’s function can be a key mechanism of pathological conditions associated with hyperdopaminergia. The first strain of gene-modified rodents with a lack of DAT were created more than 25 years ago. Such animals are characterized by increased levels of striatal DA, resulting in locomotor hyperactivity, increased levels of motor stereotypes, cognitive deficits, and other behavioral abnormalities. The administration of dopaminergic and pharmacological agents affecting other neurotransmitter systems can mitigate those abnormalities. The main purpose of this review is to systematize and analyze (1) known data on the consequences of changes in DAT expression in experimental animals, (2) results of pharmacological studies in these animals, and (3) to estimate the validity of animals lacking DAT as models for discovering new treatments of DA-related disorders.

## 1. Introduction

Dopamine (DA) is one of the most important monoaminergic neurotransmitters in the brain. The DA system is critically involved in controlling many physiological functions, including the initiation of motion, reinforcement, and motivation processes, as well as affecting emotional reactions and cognitive functions (learning, attention, memory) [1]. The key element of DA neurotransmission is undoubtedly the DA transporter (DAT). DAT, a member of the Na^+^/Cl^−^−dependent transporter family selectively expressed in dopaminergic neurons, critically regulates DA homeostasis by transporting extracellular DA into the intracellular space [2]. DAT plays a dominant role in DA clearance in the striatum; this area contains the largest amount (≈80%) of this neurotransmitter within the brain [3,4]. Thereby, DAT strictly controls the synaptic levels of DA in the mesolimbic and nigrostriatal pathways. The role of DAT in the regulation of DA metabolism in other DA pathways is less significant. The controlled pituitary prolactin secretion hypothalamic DA neurons (the tuberoinfundibular pathway) mainly release the mediator into the portal system from the median eminence [5]; however, some level of DAT seems to be expressed in the pituitary since silencing of DAT in mice results in significant pituitary hypoplasia and lactation problems (see below). At the same time it plays a significant role in the prefrontal cortex (the mesocortical pathway) [6], but the norepinephrine transporter seems to also play an important role in in DA re-uptake in this brain area [7].

A growing amount of evidence suggests that the decrease of DAT levels, as well as the polymorphisms of its gene (*Slc6a3*), are etiopathogenetic factors for the development of a wide number of DA-related disorders, including Parkinson’s disease (PD) [8,9], attention deficit hyperactivity disorder (ADHD) [9], post-traumatic stress disorder [10], drug abuse, obsessive-compulsive disorder (OCD) [11], and bipolar depression (BD) [12]. DAT is also the target for many addictive psychoactive compounds, such as cocaine, amphetamine, methamphetamine, etc. [2].

Genetic mutations in the DAT-encoding gene affect different functional parameters of the transporter: gene expression, ability to integrate into the membrane, substrates affinity, reuptake activity, transport direction, etc. These and other changes influence DA neurotransmission and contribute to the pathophysiology of CNS diseases. It is reported that numerous missense mutations of *Slc6a3* are subjected to negative selection and DAT is classified as “loss-of-function-intolerant”. This statement additionally shows the tragic consequences of DAT dysfunction [13,14,15].

Mutations in *Slc6a3* have been described in patients with DAT deficiency syndrome (DTDS). For example, some of these mutations are Ile312Phe (I312F) and Asp421Asn (D421N). These gene changes were demonstrated in a person with DTDS (Ile312Phe was inherited from his father, and Asp421Asn was a de novo mutation). As a result of these amino acid substitutions, both mutant hDATs have a reduced ability to capture DA. hDAT-I312F is characterized by low substrate affinity, high blockers affinity, and high anion conductivity. hDAT-D421N is characterized by impaired Na^+^ and Cl^−^ binding (DA co-transport ions) and constitutive leak of cations. Moreover, hDAT-D421N provokes anomalous dopamine efflux (ADE; abnormal leak of cytoplasmic DA through non-vesicular DA release by the DAT). Collectively, these events have a destructive effect on neurons [9,14].

Genetic variation of *Slc6a3* is a risk factor for autism spectrum disorder (ASD). One of the mutations is Thr356Met (T356M). This substitution occurs in highly conserved sequences of the ion binding region. The mutant hDAT-T356M is characterized by extremely low DA affinity and slow DA reuptake. Because of the occurrence of ADE, this mutation prevents the accumulation of intracellular DA. Other mutations associated with autism are Arg51Trp, Ala559Val, and ΔN336 [16,17]. ΔN336 is a rare in-frame deletion of residue Asn336 and it leads to a decrease in DA reuptake. Studies on Drosophila melanogaster demonstrated a pronounced violation of social behavior in the mutant flies [16,18].

The Ala559Val (A559V) mutation of *Slc6a3* is found not only in ASD but also in ADHD and bipolar affective disorder. It is believed that amino acid substitution leads to changes in steric interactions between transmembrane domains of hDAT and changes in transporter conformational dynamics. hDAT-A559V is characterized by increased transporter activity and the occurrence of ADE [17,19].

Some ADHD patients have Arg615Cys mutation (R615C). This mutation leads to a change in the dynamics of the DAT along the cell surface. DAT membrane transfer is an important post-translational regulatory process. It is assumed that it could be the risk factor for some CNS diseases. The R615Cys substitution is on the distal C-terminus in the region responsible for transporter relocation. Normal DAT-proteins are in special GM1/flotillin-1 enriched microdomains of the cell membrane. These microdomains limit the lateral mobility of DAT. DAT is distributed in a highly regulated manner, but hDAT-R615C constitutively recirculates throughout the cell and demonstrates insensitivity to the endocytic activation factors, possibly due to disruption of phosphorylation/dephosphorylation zones at the C-terminus of the transporter; this could be caused by the disruption of phosphorylation/dephosphorylation zones at the C-terminus of the transporter [19,20].

In a screening of the entire coding region of hDAT, a rare missense mutation, Glu602Gly (E602G), was identified in a patient with bipolar disorder. His father had this mutation as well and suffered from the same disorder. A DAT with this mutation after the translation stage is not delivered to the cell surface and does not integrate into the membrane [21].

Changes due to mutations in the *Slc6a3* gene, which correlate with CNS diseases, are found along the entire protein structure, thereby affecting various aspects of the functioning of DAT [9,15]. Each mutation in the DAT gene exhibits unique properties that ultimately lead to destructive effects on the nervous system [14]. It is worth noting that a relationship has been found between the type of mutation and the response to therapeutic agents. This relationship has been established for ADHD and PD. This fact needs to be verified in relation to other diseases in order to improve the effectiveness of treatments [15].

## 2. Genetically Modified Animals Affecting Function of DAT

Genetically modified animals deficient in DAT (knockouts (KO) or knockdowns (KD)) are still one of the most used models for studying DA functions. Summary information about the stocks and strains is presented in Table 1.

According to Table 1, the first mouse strain lacking DAT was developed more than 25 years ago [22]. Studies on mice with decreased DAT expression have significantly expanded our understanding of the basic principles of DA neurotransmission, the mechanisms of action of various psychotropic drugs, and the interaction of the most important neurotransmitters of CNS, as well as the pathophysiological mechanisms of DA-related disorders. However, certain questions can be more reliably addressed in transgenic rats [32]. The first gene-modified rats that lack of DAT were introduced to scientists in 2016–2018 [29,30]. The greater sizes of rats and their brains allow for a number of practical benefits, especially in relation to surgical (mainly, neurosurgical) techniques [33,34]. Moreover, there are strong differences in neuroanatomical and neuroprotein pattern expression between rats and mice, giving rise to rats’ richer behavior and demonstrating more robust and reproducible performance in cognitive tasks [32,34].

## 3. Impact of DAT Deletion on Neurotransmission and Neuroanatomy

Striatal DA neurotransmission is dysregulated in both DAT-KO rats and mice. The lack of DAT expression is known to lead to approximately a 5–7-fold increase in extracellular DA levels in the striatum (Str), one of the essential structures of CNS, involving both nigrostriatal (transmitting DA from substantia nigra pars compacta (SNc) to the caudate nucleus and putamen) and mesolimbic (transmitting DA from the ventral tegmental area (VTA) to the nucleus accumbens (NAcc)) pathways [24,30,35,36]. DA persists for a longer period in the synaptic cleft, and consequently, extracellular clearance of DA, which is mainly driven through diffusion, is delayed by almost 40–300 times compared to WT controls [22,30,37,38,39,40]. Concurrently, the intracellular tissue content of striatal DA is decreased by 13–20-fold, suggesting a crucial contribution of DAT in the sustainment of intracellular stores of DA [24,30,35,36,40]. Additionally, these results are also supported by a reduction of striatal mRNA and the alterations of protein expression and phosphorylation of tyrosine hydroxylase (TH), the rate-limiting enzyme for DA biosynthesis [23,30,35,41]. Specifically, TH mRNA levels are only marginally reduced, while protein levels are reduced by almost 90%; notably, its immunoreactivity is almost undetectable in several striatal projections [41]. Decreased intracellular DA pool apparently leads to a reduced stimulated DA release [24,37,38]. Nonetheless, it should be noted that the lack of DAT does not result in changes in intracellular DA transport in vesicles [40,41]. Additionally, one more feature of DA tone in DAT-KO rodents is the dearth of diurnal variation observed in wild-type (WT) animals [42].

DA overflow in DAT-KO and DAT-KD animals causes a permanent activation of postsynaptic D1- and D2-like DA receptors (D1R and D2R, respectively) [22,29,43,44], which results in their down-regulation [30,43,45,46]. This also leads to a decreased expression and function of presynaptic D2R in DAT-KO animals [22,42,47]. Notably, in DAT-KD mice, only the decrease of presynaptic D2R expression was shown [24]. At the same time, down-regulation of DA autoreceptors was not detected when DAT expression was decreased in adult animals (tetracycline-inducible DAT-KD), an aspect that may point to long-term mechanisms of their down-regulation [28].

Increased DA concentration in DAT-KO animals is associated with its intensified degradation by catechol-O-methyltransferase (COMT) and monoamine oxidase (MAO), which results in increased levels of such DA metabolites as 3-methoxytyramine, 3,4-dihydroxyphenylacetic acid, and homovanillic acid [30,35,40]. Interestingly, COMT inhibition does not affect the rate of synaptic DA clearance while MAO inhibition prolongs DA half-life [30,38]. Considering that MAO is predominantly involved in the oxygenation of intracellular DA, we might speculate that homeostasis of synaptic DA strongly depends on the pool of DA synthesis de novo in the case of DAT hypofunction. The other possible explanation of COMT inhibition’s “ineffectiveness” is that action of tolcapone administration on DA levels was measured in the striatum. DA metabolism seems to primarily rely on COMT in PFC but not in the striatum [48].

Structurally, DAT-KO animals show a reduced volume of the striatum followed by a concurrent volume increase in other important regions, such as the PFC and cerebellum [49]. Striatal volume loss in mice is mainly caused by a decreased density of GABAergic interneurons and raised markers of neurodegeneration (e.g., hyperphosphorylated tau protein) [50,51,52,53]. Notably, this negative correlation between striatal and cerebellar volume areas points out a potential neurodevelopmental compensation [49].

In addition, the decreased DAT activity leads to biochemical and structural changes that also affect other neurotransmitter pathways. Indeed, DAT-KO animals showed a decreased density of GABAergic neurons, as well as decreased concentrations of anandamide and serotonin [30,52]. Moreover, elevated numbers of neurodegeneration markers, such as hyperphosphorylated tau protein, that are also associated with dyskinesia manifestation, have been revealed in DAT-KO mice striatal samples [50,51,52,53].

Another brain area potently altered by DAT deletion or hypofunction is PFC, where monoamine alterations play a crucial role in the development of various neuropsychiatric disorders [54]. Notably, an increased and prominent hyperconnectivity has been observed in the cortico-striatal circuit in DAT-KO compared to WT animals [49]. These alterations in the PFC could lead to working memory impairments that might be caused by several different pathways and mechanisms. For example, Leo et al. found downregulation in the neurotrophin BDNF mRNA and protein levels, as well as the downstream pathway, that involve high-affinity receptor TrkB [30]; pro-inflammatory processes in the PFC of female rats lacking DAT was also seen in another study, suggesting a prominent role in neurodegeneration activity [55]; and, recently, Targa et al. found significant dysregulation of AMPA receptor trafficking through an altered endosomal regulation [56].

All these morphological and functional variations also induce neurophysiological and synaptic plasticity disruptions such as, on the one hand, increased long-term potentiation (LTP) in the cortico-striatal synapses that is correlated with a decrease in PSD-95 concentration [57,58] and, on the other hand, LTP deficiency in the 5th layer of cortical pyramidal neurons [6,59]. At the same time, a weakening of long-term depression in the hippocampus was also seen [57]. Additionally, changes in cortical and striatal power spectra and interareal coherence were detected [60]. Proteomic analysis revealed modifications in striatal proteins expression that were closely related to learning and memory mechanisms (i.e., synaptic transmission, axodendritic transport, and DA-binding processes) [61].

In general, disruption of the DAT function provokes significant DA neurotransmission impairments affecting almost all its stages, highlighting the key role of DAT in the maintenance of DA homeostasis and the dramatic neurobiological changes in the CNS, which mostly concern the cortico-striatal system.

## 4. Impact of DAT Deficiency on Animal Physiological Phenotype

Unlike DAT-KD [24] animals, DAT-KO [22,30,62] are characterized by reduced body weight. Even though mutant animals do not show any propensity to die at birth, DAT-KO mice (but not rats) have increased mortality [22,50,63]. However, it should be noted that genetic background can affect DAT-KO mice’s rate of survival [64]. DAT-KO mice show a higher mortality rate at all ages in comparison to heterozygotes and WT littermates. Just before death, mutant mice are characterized by loss of hyperactivity, tremor appearance, rapid weight loss, and pronounced dorsal kyphosis [50], and, correspondingly, reduced bone strength [64]. Intriguingly, premature death was prevented in DAT-KO mice gene therapy by expressing DAT selectively in DA neurons and terminals through gene therapy [65]. Furthermore, DAT silencing was found to affect colon peristaltic in mice [66]. In addition, DAT-KO mice display reduced breath rate, body temperature, and rod sensitivity [67,68]. Deletion of DAT in mice is also accompanied by decreased natural killer cell activity and mitogen-induced cytokine responses [69]. In contrast, LPS-induced cytokine production by macrophages was enhanced in DAT-KO mice [69]. Aberrant immune reaction, as well as reduced angiogenesis, can cause decreased tumor growth [70]. Even though little is known about the effects of DAT-deficits, it is obvious that DAT depletion seriously affects many body organs. Further studies aimed at analyzing these actions are warranted. In addition, these effects should be considered for a correct interpretation of behavioral experiment data aiming at studying central nervous system functioning.

## 5. Behavioral Phenotype of Animals with DAT Hypofunction

The most pronounced behavioral feature of DAT-deficient animals is locomotor hyperactivity [22,23,30,31,37,71,72,73,74,75]. When animals are placed in their home cages, this hyperactivity appears mainly during the dark phase [30], and it is exacerbated in a new environment. Such hyperactivity is observed in a new environment only in the case of partial deficiency of DAT (DAT-KD and DAT-LE mice) [24,25,27,76]. Other aberrant motor reactions are observed in DAT-KO rodents: increased level of stereotypies [31,74,77,78], a reduced fore- and hind-limb mean stride length [50,53] (for controversial see [74]), and impaired motor coordination [53].

The impact of DAT deficit on Negative Valence Systems has been studied very intensively. In several studies, the reduction of depression-like behavior development [71,79,80,81] and decreased anxiety [82,83,84] were observed in DAT-KO rodents. Notably, the selective decrease of DAT expression in NAcc of adult mice resulted in the same behavioral changes [85]. It should also be considered that ablation of DAT is associated with altered behavioral reactions to stress [86]. It should also be noticed that some scientific groups reported controversial results regarding this topic. For example, Takamatsu et al. failed to find any difference between DAT-KO and WT mice in a tail suspension test [87], and no signs of decreased anxiety were shown in the elevated plus maze in DAT-KD rats from Prof. Spanagel’s group [72]. 

Much less is known about the impact of DAT deficits on sensitivity to reward stimuli (Positive Valence System). According to some studies performed on DAT-KO mice, silencing of DAT was associated with increased sucrose solution consumption [71] and positive bias toward a hedonically positive tastant [80]. In addition, Pecina and colleagues reported that DAT-KD mice have greater incentive performance for a sweet reward [88]. At the same time, DAT-KO and DAT-KD rats were demonstrated not to have a preference for sweet solutions [72,89]. Moreover, Mallien and colleagues found less preference for saccharin in the two bottle test in DAT-KO rats [74]. DAT-KO mice also exhibited a stronger rewarding response to morphine compared with control littermates [79].

During experiments with food reinforcement, the lack of DAT does not affect the operant conditioning of DAT-KO and -KD mice [44,90,91,92,93]. We further supported these observations in DAT-KO rats trained to perform lever presses [94,95]. DAT deficiency-associated hyperdopaminergia seems to seriously affect rodent behavior when conditions of schedules were changed. In this way, DAT-KO mice failed to change the temporal pattern of their responses in either fixed-interval or peak-interval timing procedures [93]. In addition, these mice are characterized by enhanced resistance to extinction [90].

It is well established in experiments in both humans and rodents that DA levels contribute to cost-benefit analysis. High DA levels are associated with a preference for “high-cost” reactions [96,97,98]. Vice versa, low DA levels are correlated to the preference for “low-cost” reactions [99,100,101]. In full concordance, DAT-KD mice are characterized by a preference for “high-cost” responses in the “closed economy” paradigm [91]. DAT-KD mice also earned more reinforcers than WT littermates under the progressive ratio paradigm [44]. However, we failed to reproduce these results in DAT-KO rats [95]; indeed, mutant and control rats acquired the same number of reinforcers. However, the local response rate dynamics were dramatically changed in the DAT-KO rats: progressive increase of ratio was accompanied by a decrease in local response rate in control animals; on the contrary, in DAT-KO littermates, the response rate was gradually increased as the required number of responses to obtain a reward was growing.

Considerable efforts have been made at studying the impact of DAT deficit on cognitive functions. The full lack of DAT in rodents is accompanied by spatial memory deficits in the Morris water navigation task, 8-arm, Hebb–Williams, T-, H-, and Y-mazes [30,57,77,102,103,104,105]. However, Chang et al. failed to find memory deficits in DAT-KD mice in the Morris water navigation task and Y-maze [106]. DAT-KO and DAT-KD learning and memory deficits were also revealed in a novel object recognition test [72,106]. Although the lack of DAT does not seem to affect simple Pavlovian conditioning [61,72,92], DAT-KO rats were incapable of learning new stimulus-response associations [61], while DAT-KO mice performed less avoidance in the Conditioned Avoidance Responding Test than WT littermates [87]. Similarly, aberrant Pavlovian-to-instrumental transfer was revealed in DAT-KD mice [92]. Part of the learning deficits associated with DAT silencing might be explained by their locomotor hyperactivity; however, the role of DA in the processes of assigning incentive salience to stimuli plays a key role in this aspect [107]. Under hyperdopaminergic conditions, this process might become aberrant and result in learning deficits as well as a reduction of stimulus control. The reported results of experiments on sensorimotor gating indirectly support this speculation on DAT-deficits association with impairment of sensory information filtration. Thus, decreased pre-pulse inhibition (PPI) was demonstrated in both DAT-KO mice [108,109,110,111,112,113] and rats [30], but not in DAT-KD mice [114]. It should also be noted that DAT-KO animals are characterized by increased amplitude of startle response [30,109,110]. However, Kurzina et al. reported decreased amplitude in DAT-KO rats [60]. Additionally, olfactory discrimination deficits were demonstrated in DAT-KO mice [115].

Rodents lacking DAT also show some atypical social behavior traits. For instance, female DAT-KO mice are characterized by a deficit of maternal behavior [64,71]. This deficit can be explained by either locomotor hyperactivity or decreased prolactin release [62]. At the same time, it is not quite clear whether the lack of DAT affects social interaction. On the one hand, some research groups observed impaired social behavior in DAT-KO and DAT-KD rats [72,74]; on the other hand, Cinque and colleagues found intact social behavior in DAT-KO [89]. The different methods used in these studies (social interaction with an unfamiliar social partner vs Social Preference Test) can explain the discrepancies between them. Interestingly, DAT-KO mice retain the ability to establish social hierarchies, but the DAT deficit was accompanied by increased rates of reactivity and aggression [116]. However, little is known about the sexual behavior of DAT-KO animals; only Sanna et al. reported that DAT-KO rats have a more rapid acquisition of stable sexual activity levels and higher levels of sexual motivation and activity [117].

In summary, we can conclude that lack of DAT is associated with significant hyperactivity and impairment of motor function control, mild cognitive (spatial memory and learning) deficits, increased motivation for reward, aberrant cost-benefit analysis, and few changes of social behavior (most reliable one being the deficit of maternal behavior). However, data on behavioral methods in cases of DAT silencing should be considered with caution since hyperactivity associated with the lack of DAT can affect the reported results. Moreover, we hypothesize that hyperdopaminergia associated with DAT silencing might result in depleted behavioral flexibility. A number of scientists working with animals lacking DAT reported that these animals’ behavior is characterized by perseverative patterns of behavior. For example, hyperactivity of both DAT-KO mice and rats manifests in exhibited non-focal preservative patterns of locomotion [49,53,108]. Rodriguiz et al. reported that the aggressivity of DAT-KO mice can be partly caused by stereotyped and perseverative patterns of their social responses [116], features which are also shown in DAT-KO rats [89].

## 6. DAT Deficient Animals as Models in Experimental Neuropsychopharmacology

### 6.1. DTDS

Currently, DAT-KO mice have proven themselves to be the best animal model for Dopamine Transporter Deficiency Syndrome (DTDS) [65]. DTDS is an inherited DA “transportopathy” resulting from missense variants of the *Slc6a3* gene. Depending on the type of mutation, the patients might have symptoms of ADHD, atypical parkinsonism, or autism [15]. The classic manifestation of DTDS is a hyperkinetic movement disorder with onset in infancy and progression to severe parkinsonism in early childhood. Another manifestation of the disease is an atypical DTDS, which is characterized by manifestation in later childhood with a relatively milder course of the disease [118,119]. It was shown that DAT dysfunction may consist in the loss of primary DAT function, which leads to the absence or a strong decrease in DA uptake, a decrease in DAT binding to the cell surface, a decrease in the affinity of DAT for DA, a decrease in DA recognition by DAT, a decrease in DAT expression with a predominance of an excess of non-glycosylated DAT [118].

DAT-KO mice reproduce the main clinical features of DTDS patients, which have a tendency to develop recurrent hyperkinesis from an early age, development of motor deficits, loss of the ability to move, hyperactivity, striatal neurodegeneration, high mortality, and a patient-like change in the concentration of DA metabolites [65]. The validity of this model allows testing of the potential variant of DTDS therapy, for example, using gene therapy. Using a dual combinatorial AAV-based gene therapy approach, aimed at the restoration of native *Slc6a3* gene expression, it was possible to ensure stable expression of healthy DAT in dopaminergic neurons of the midbrain and striatum. This method of therapy led to the normalization of DA signaling and markedly changed the behavioral phenotype of mice; for example, the development of motor disorders that led to death was completely prevented [65].

### 6.2. States Associated with Increased Levels of DA

Increased DA neurotransmission has been hypothesized to contribute to the pathogenesis of several mental illnesses and conditions. Animals with a lack of DAT are thought to model some signs and symptoms of ADHD (for review see [120]), schizophrenia [121], maniac phases of BD [76], protracted abstinence [29], and dopamine dysregulation syndrome (Sukhanov, Volnova and Gainetdinov, unpublished). However, it should be considered that (1) the accurate impact of DAT hypofunction in the pathogenesis of the listed above diseases is partially controversial and (2) with the exception of inducible DAT-KD mice, these animals have permanent hyperdopaminergia as soon as DA structures in the brain are formed in the prenatal period.

To illustrate the limited usefulness of the approach to the DAT-depleted rodents as the models of specific mental diseases, we collected results of pharmacological tests with compounds mitigating their increased locomotor activity (Table 2). Studies demonstrate that pretreatment with drugs used to control ADHD symptoms, such as amphetamine and methylphenidate, resulted in a dose-dependent reduction of hyperactivity in DAT-KO and -KD rodents and increased locomotor activity of WT and HT littermates [24,30,71,77]. Fluoxetine decreased the locomotor activity of DAT-KO rodents without any effect on control animals’ motor behavior [77]. However, the action of the other anti-ADHD agent, atomoxetine, seems to be non-selective. Administration of atomoxetine resulted in decreased locomotor activity of both WT and KO rats [72]. Of course, the list of the agents mitigating hyperactivity is not limited only to the ones mentioned above, and many of them cannot be used to treat ADHD even theoretically. In summary, we suggest employing DAT-deficit rodents in the first place to model behavioral correlates of hyperdopaminergia but not some specific pathology.

### 6.3. States Associated with Decreased Levels of DA

In case of lack of DAT, as mentioned above, the vesicular storage of DA is depleted. DA release is therefore strongly dependent on its de novo synthesis mediated by tyrosine hydroxylase (TH). Thus, animals with DAT silencing are very sensitive to the actions of irreversible TH inhibitor alpha-methyl-para-tyrosine (aMPT). Following its administration, synaptic DA disappears in DAT-KO animals [38,40,122,123]. Behaviorally, the abrupt DA depletion is accompanied by striking akinesia and catalepsy [122,123,124]. In summary, aMPT-treated animals (DA deficient DAT-KO—DDD rodents) can be considered as a model of DA deficiency associated with PD [122,124]. Pretreatment with DA precursor L-DOPA and DA receptor agonists results in locomotor activity recovery and/or elimination of catalepsy [123,124]. Primarily, the mutual activation of both D1- and D2-R is thought to be required for these effects. However, we demonstrated that activation of striatal D1R expressing medial spiny neurons by phosphodiesterase 10A inhibitors seems to be sufficient for the recovery of motor functions in DDD rats [123]. Administration of amphetamine-like psychostimulants is also able to revert aMPT effects on locomotor activity in DDD mice [124]. Non-dopaminergic mechanisms are supposed to be responsible for psychostimulant action [124]. Additionally, DA depletion in mice is accompanied by suppression of slow-wave sleep and REM sleep disappearance [125]. In this case, treatment with D2-(but not D1-) receptor agonists can recover REM sleep in these mice [125].

**Table 2:** Pharmacological agents decreasing locomotor hyperactivity in rodents with lack of DAT.

Drug	Mechanism of Action	Animals	Doses	Administration Route	Administration Schedule	Number of Animals per Group	Influencing WT Activity	Reference
Nicotine	NAchR agonist	DAT-KO mice F1	0.5 and 1 mg/kg	s/c	before testing	16–24	**↑**	[46]
		DAT-KO mice	1 and 3 mg/kg	i/p	before testing	8–12	-	[113]
		DAT-KD C57BL6/J mice	40 mg/kg/day	s/c, by mini-osmotic pumps	for 26 days	27–30	-	[126]
Choline	α7 agonist	DAT-KO mice F1	5 mg/kg	s/c	before testing	12	-	[46]
Epibatidine + Choline	β2 agonist + α7 agonist	DAT-KO mice F1	0.5 mcg/kg + 0.5 mg/kg	s/c	before testing	8	-	[46]
β-phenethylamine	TAAR agonist	DAT-KO mice C57BL/6J x 129/SVJ	10, 30, 50, 70, 100 mg/kg	i/p	30 min before testing	10–15	**↑**	[127]
Methylphenidate	DAT and NET inhibitor	DAT-KO mice 129/C57	30 mg/kg	i/p	before testing	8	**↑**	[77]
		DAT-KO rats Wistar Han	1.2, 2.5, 5 mg/kg	i/p	before testing	6–19		[30]
Haloperidol	D2R antagonist	DAT-KO mice 129/C57	0.2 mg/kg	i/p	before testing	6	**↓**	[77]
		DAT-KO mice C57BL/6	0.15, 0.2, 0.3 mg/kg	s/c	30 min before testing	10–15		[71]
		DAT HET mice C57BL/6	0.1, 0.15, 0.2, 0.3 mg/kg	s/c	30 min before testing	10–15		[71]
		DAT-KO rats Wistar Han	0.5 mg/kg	s/c	before testing	6–19		[30]
		DAT HET rats Wistar Han	0.5 mg/kg	s/c	before testing	4		[30]
Clozapine	D2-/5-HT2A antagonist	DAT-KO mice C57BL/6	2 and 3 mg/kg	s/c	30 min before testing	10–15	**↓**	[71]
		DAT HET mice C57BL/6	1, 2, 3 mg/kg	s/c	30 min before testing	10–15		[71]
Raclopride	D2R antagonist	DAT-KO mice C57BL/129SvJ	0.1 mg/kg	i/p	10 min before testing	9–18	-	[108]
SCH23390	D1R antagonist	DAT-KO mice C57BL/129SvJ	0.01 mg/kg	s/c	10 min before testing	9–18	-	[108]
Cocaine	DAT, SERT and NET inhibitor	DAT-KO mice 129/C57	40 mg/kg	i/p	before testing	8	**↑**	[77]
Amphetamine	DAT, SERT and NET inhibitor, DA and 5-HT receptors agonist	DAT-KO mice C57BL/6	1, 3, 10 mg/kg	s/c	before testing	8–15	**↑**	[128]
		DAT HET mice C57BL/6	1 mg/kg	s/c	before testing	11–16		[128]
		DAT-KO mice 129/C57	0.75 mg/kg	i/p	?	?		[77]
			2 mg/kg	i/p	before testing	8		[77]
		DAT-KO rats Wistar Han	1, 2, 3, 4 mg/kg	i/p	before testing	6–19		[30]
		DAT-KD rats F344	1 and 2 mg/kg	i/p	30 min before testing	7–10		[72]
		DAT-KO mice F1	3 mg/kg	i/p	30 min before testing	7–11		[129]
		DATKO mice C57Bl/6J	100 μM/0.5 μL/side	bilateral PFC infusion	before testing	6–8		[130]
		DAT-KD mice 129 SvyJ	1, 2, 3 mg/kg	i/p	before testing	6–8		[24]
Fluoxetine	SERT inhibitor	DAT-KO mice 129/C57	20 mg/kg	s/c	before testing	6	-	[77]
		DAT-KO mice C57BL/6	20 mg/kg	s/c	before testing	5		[128]
		DAT-KO mice C57Bl/6J	20 mg/kg	s/c	before testing	6–12	?	[130]
Quipazine	5-HT 2A and 5-HT3 agonist	DAT-KO mice 129/C57	3 mg/kg	i/p	before testing	6	-	[77]
			0.5 mg/kg	i/p	?	?		[77]
5-HTP	serotonine precursor	DAT-KO mice 129/C57	50 mg/kg	i/p	before testing	6	**↓**	[77]
			10 mg/kg	i/p	?	?	-	[77]
L-Tryptophan	serotonine precursor	DAT-KO mice 129/C57	100 mg/kg	i/p	before testing	6	-	[77]
			10 mg/kg	i/p	?	?		[77]
RO5203648	partial TAAR1 agonist	DAT-KO rats Wistar Han	3 mg/kg	i/p	before testing	6–19	-	[30]
RO5166017	partial TAAR1 agonist	DAT-KO mice C57BL/129SvJ	0.5 and 1 mg/kg	i/p	before testing	7–8	-	[131]
Apomorphine	DA receptors agonist	DAT-KD mice 129 SvyJ	0.1, 0.5, 1, 2 mg/kg	s/c	before testing	6–8	**↑**	[24]
Quinpirole	D2/D3 agonist		0.1, 0.5, 2, 6, 20 mg/kg	i/p	before testing	6–8	**↑**	[24]
MDMA	DAT, SERT and NET inhibitor, DA and 5-HT receptors agonist	DAT-KO mice C57BL/129SvJ	20 mg/kg	i/p	10 min before testing	7–11	**↑**	[132]
SL 327	MEK inhibitor	DAT-KO mice C57BL/129SvJ	100 mg/kg	i/p	before testing	11	-	[133]
Reboxetine	NET inhibitor	DAT-KO mice F1	5 and 10 mg/kg	i/p	30 min before testing	6–18	-	[129]
Atomoxetine		DAT-KD rats F344	1 and 3 mg/kg	i/p	30 min before testing	7–11	**↓**	[72]
Desipramine		DATKO mice C57Bl/6J	25 mg/kg	i/p	before testing	6–12	?	[130]
			4 µg/0.5 µL/side	bilateral PFC infusion				
U99194	D3 antagonist	DAT-KO mice F1	30 and 60 mg/kg	i/p	30 min before testing	5–7	-	[129]
SB-277011A	D3 antagonist	DAT-KO mice F1	3, 10, 30 mg/kg	i/p	30 min before testing	5–7	-	[129]
LY-341495	MGluR2 antagonist	DAT-KO rats N157K	1, 3, 10 mg/kg	i/p	30 min before testing	6–9	-	[72]
SB 224289	5-HT1B antagonist	DAT-KO mice C57BL/129SvJ	20 mg/kg	i/p	60 min before testing	10	-	[134]
M100907	5-HT2A antagonist	DAT-KO mice C57BL/129SvJ	0.3 and 1 mg/kg	s/c	30 min before testing	10–17	-	[135]
Aniracetam	AMPA positive allosteric modulator	DAT-KO mice C57BL/129SvJ	20 and 50 mg/kg	i/p	before testing	8–10	?	[136]
CX516		DAT-KO mice C57BL/129SvJ	100 mg/kg	s/c	before testing	6–11	?	[136]
CX546		DAT-KO mice C57BL/129SvJ	50 and 70 mg/kg	s/c	before testing	6–11	?	[136]
CX672		DAT-KO mice C57BL/129SvJ	1 mg/kg	i/p	before testing	?	?	[136]
CX776		DAT-KO mice C57BL/129SvJ	3 mg/kg	s/c	before testing	?	?	[136]
Pregnenolone	GABA A and NMDA allosteric modulator	DAT-KO mice C57BL/6	30 and 60 mg/kg	i/p	before testing	10–15	-	[109]
Donepezil	cholinesterase inhibitor	DAT-KO mice C57BL/6	1 and 3 mg/kg	i/p	20 min before testing	?	**↓**	[137]
Tacrine		DAT-KO mice C57BL/6	3, 10, 30 mg/kg	i/p	20 min before testing	?	**↓**	[137]
VU0152100	M4 positive allosteric modulator	DAT-KO mice C57BL/6	1 mg/kg	i/p	20 min before testing	5	?	[137]
AM404	Anandamide reuptake inhibitor	DAT-KO mice F1	0.3, 1, 3 mg/kg	i/p	before testing	8	-	[52]
VDM11			2 and 5 mg/kg	i/p	before testing	6–8	-	[52]
AA5HT	FAAH inhibitor		2 and 5 mg/kg	i/p	before testing	6–8	-	[52]
Valproic acid	Na channels blocker, GABA enhancer	DAT-KD mice 129 SvyJ	100 mg/kg	i/p	60 min before testing	14	-	[114]
		DAT-KO mice C57BL/129SvJ	300 mg/kg	i/p	before testing	8	**↓**	[138]
LiCl	GSK-3 inhibitor	DAT-KO mice C57BL/129SvJ	50, 100, 200 mg/kg	i/p	before testing	9–12	?	[138]
SB 216763			3, 5, 10 mg/kg	i/p	before testing	6–8	?	[138]
Indirubin			10 and 20 mg/kg	i/p	before testing	8–11	?	[138]
Alsterpaullon			3, 5, 10 mg/kg	i/p	before testing	8–10	?	[138]
TDZD			30 mg/kg	i/p	before testing	7	?	[138]
ABM300	CB1R allosteric modulator	DAT-KO mice C57Bl/6J	10 mg/kg	i/p	30 min before testing	10–13	-	[139]
Nepicastat	DBH inhibitor	DAT-KO mice C57Bl/6J	40 mg/kg	i/p	before testing	6–12	?	[130]
			4 µg/0.5 µL/side	bilateral PFC infusion				

↑—increase of locomotor activity; ↓—decrease of locomotor activity; - —no effect; ?—effect did not described in the study; s/c—subcutaneous; i/p—intraperitoneal.

## 7. Conclusions and Future Directions

Summing up, the phenotypic features of DAT-deficient animals are frequently related to pathogenesis and symptoms of such DA-related disorders as schizophrenia, ADHD, drug addiction, etc. According to our opinion (Table 3), described in the present review, DAT-deficient animals have some but limited usefulness as models of neuropsychiatric disorders. The division of lack of DAT into nigrostriatal and mesolimbic pathways is, therefore, the most obvious way to create a new type of deficiency-based model. Similar region-selective approaches should be applied for DAT overexpressing rodents since only mice with global overexpression of DAT are currently available [140]. The second direction is the selective depletion of DAT in particular DA neurons. It is well-known that most of striatal GABA-ergic medium spiny neurons (MSNs) belong to either D1R or D2R expressing populations [141,142]. Distinct populations of MSNs give rise to direct and indirect pathways in the frame of both nigrostriatal and mesolimbic pathways. There are four pathways that originate from MSNs that seem to play different roles. We suggest that selective hyperdopaminergia allows us to better understand the role of DA in these pathways in normal conditions and in the pathogenesis of neuropsychiatric disorders.

## Figures and Tables

**Table 1:** Rodent strains lacking DAT.

Rodent Specie	Stocks and Strains	% of Dopamine Transporter Expression Decrease	Methods and Selectivity	Reference
Mice	C57/B6Jx129/Sv/J and C57/B6J	100%, DAT-KO	in vivo homologous recombination	[22]
	C57/BLy6J	100%, DAT-KO		[23]
	129 Sv/J	90%, DAT-KD		[24]
	C57/BL6	45–50%, DAT lower expresser	Knock-in of hemagglutinin epitope in EL2	[25]
	C57BL/6J	Variable (inducible DAT-KD)	Intra-accumbal delivery of DAT shRNA-expressing lentiviral vectors	[26]
	BALB/c		Intraventricular local nonviral RNA interference	[27]
	C57BL6/J		Tetracycline inducible system	[28]
Rats	Fischer 344	Less than 25%, DAT-KD	N-ethyl-N-nitrosourea-induced spontaneous mutation	[29]
	Wistar Han	100%, DAT-KO	Zinc finger nuclease technology	[30]
	Wistar		CRISPR/Cas9 technology	[31]

DAT—dopamine transporter; DAT-KO—DAT knockout; DAT-KD—DAT knockdown.

**Table 3:** Validity of animals with DAT hypofunction as the model of neuropsychiatric disorders.

Model	Face Validity					Predictive Validity				
	Schizophrenia	ADHD	Mania	Drug Dependence	Parkinson Disease	Schizophrenia	ADHD	Mania	Drug Dependence	Parkinson Disease
DAT KO	+	+	+	?		+	++	+	?	
DAT KD	-	+	+	+		-	+	++	?	
DDD					++					++

DAT-KO—dopamine transporter knockout; DAT-KD—dopamine transporter knockdowns; DDD—dopamine deficient DAT-KO; ++—high validity; +—moderate validity; - —no validity; ?—no information.

## Data Availability

Not applicable.
